# Combining a Modified Emergency Severity Index Score with Chief Complaints - Validation of a New Allocation Algorithm for Integrated Emergency Centers

**DOI:** 10.1016/j.acepjo.2026.100534

**Published:** 2026-11-05

**Authors:** Domagoj Schunk, Alina Balandin, Jan-Niclas Kersebaum, Milda Aleknonyte-Resch, Arvid Lepsien, Hans-Jörg Busch, Florian Ketels, Sascha Thielke, Sebastian Wolfrum

**Affiliations:** 1 Interdisciplinary Emergency Department and Pediatric Emergency Department, University Medical Center Schleswig-Holstein, Kiel Campus, Germany; 2 Acute and Emergency Medicine Section of the German Society for Medical Intensive Care and Emergency Medicine (DGIIN e.V.), Germany; 3 German Society for Emergency Medicine (DGINA e.V.), Germany; 4 Department of Anesthesiology and Operative Intensive Care Medicine, University Medical Center Schleswig-Holstein, Kiel Campus, Germany; 5 Christian-Albrechts-University, Institute of Business Administration, Kiel, Germany; 6 Institute for Multimedia and Interactive Systems, University of Lübeck, Lübeck, Germany; 7 Department of Computer Science, Christian-Albrechts-University, Kiel, Germany; 8 University Emergency Center, University of Freiburg, Freiburg, Germany; 9 University Medical Center Schleswig-Holstein IT Services GmbH, Kiel, Germany; 10 Interdisciplinary Emergency Department, University Medical Center Schleswig-Holstein, Lübeck Campus, Germany

**Keywords:** central point of assessment (CPA), integrated emergency center (IEC), emergency severity index (ESI), triage, acute outpatient clinic (AOC), clinical decision support system, crowding

## Abstract

**Objectives:**

Current health policy reforms in Europe aim to synchronize emergency departments and acute outpatient clinics (AOC)/primary care services through the establishment of Integrated Emergency Centers (IECs). A central challenge of this model is the upstream triage at a central point of assessment (CPA). To date, there is a lack of standardized concepts for a single-stage assessment conducted by emergency-qualified staff that also facilitates cross-sectoral allocation. To address this gap, this study introduces the optimized leading-symptom-weighted modified Emergency Severity Index (L-mESI opt.) triage instrument. This tool aims to safely distribute the clinical workload between emergency departments (EDs) and AOCs by linking a modified emergency severity index (mESI)—omitting vital signs—with the patient’s chief complaint (CEDIS).

**Methods:**

A retrospective analysis of walk-in emergency patients was conducted at two university hospitals in 2024 (training dataset: n = 26,845). Outpatient treatment rates were determined for combinations of chief complaints and mESI categories. These probabilities informed an optimized allocation algorithm (L-mESI opt.) via receiver operating characteristic (ROC) analysis and the maximum Youden Index. The model was verified using a separate validation dataset (n = 11,512).

**Results:**

The pretest probability for AOC allocation was identified at 74%. The optimized L-mESI-opt. algorithm correctly allocated 54.2% of patients to the AOC, the ED workload was reduced by 46.9%. Compared with standard ESI-based steering, the L-mESI-opt. significantly improved sensitivity for inpatient care from 54.5% to 69.5% and achieved a negative predictive value of 85.4%. Although 14.6% of AOC-allocated patients required subsequent ED admission (“Upstream”) in the validation set, critical outcomes remained minimal (observation unit transfer: 0.3%; mortality: 0.1%). Validation confirmed consistent quality and safety.

**Conclusion:**

L-mESI opt. model demonstrates the potential to effectively reduce ED workload while maintaining high patient safety in a simulated environment. These results show that its adaptability could allow for broad application in IECs. However, the successful implementation of such an algorithm will likely depend on close spatial and organizational links between EDs and AOCs to manage bilateral patient flow efficiently. Prospective clinical trials are warranted to confirm these findings in real-world settings.

**The Bottom Line**

This study investigated whether combining a modified emergency score with patient-reported chief complaints can safely streamline emergency department workflows. By simulating this new optimized leading-symptom-weighted modified emergency severity index algorithm on over 38,000 patients, the system safely redirected nearly half of the walk-in population to an acute outpatient clinic. Importantly, it achieved this massive workload reduction while maintaining high patient safety, lowering critical missed cases to less than 1%. These findings suggest that integrated emergency centers can significantly reduce crowding without risking patient care, provided that the outpatient clinic and emergency department share the exact same physical location.

## 1 Introduction

### 1.1 Background

The upcoming emergency reform in Germany mandates the establishment of Integrated Emergency Centers (IECs), in which the Central Point of Assessment (CPA) plays a pivotal role. The CPA is intended to steer patients to the appropriate care structure using standardized triage procedures.^1^, ^2^

### 1.2 Importance

Recently, a widely used software solution for structured medical initial assessment was evaluated in Germany, revealing an overestimation of care urgency in nearly two-thirds of patients.^3^ Furthermore, a potentially dangerous underestimation of urgency occurred in 2.7% of the cases. Another analysis utilized a traditional emergency severity index (ESI) system in an IEC to categorize patients simultaneously by urgency and appropriate care sector. This reduced the proportion of low-risk patients in the ED, although 8.7% of patients had to be redirected from the on-call practice/AOC back to the ED.^4^

### 1.3 Goals of This Investigation

In a recent study, our working group demonstrated that integrating Canadian Emergency Department Information System (CEDIS) chief complaints into the Manchester Triage System (L-MTS opt.) enables safe patient steering.^5^ However, the MTS and the globally prevalent ESI operate on fundamentally different triage paradigms. Whereas MTS relies on rigid syndromic flowcharts, ESI strongly depends on predicting resource utilization. Therefore, it remains unknown whether a symptom-based optimization strategy is system-agnostic. The primary objective of this study is to investigate whether this concept can be successfully translated into a resource-predictive score by developing the optimized leading-symptom-weighted modified emergency severity index (L-mESI opt.). Proving efficacy across fundamentally distinct triage philosophies is a mandatory prerequisite before recommending such steering algorithms as a universal standard for future IECs.^5^

A critical bottleneck in current ED triage systems is the time-consuming acquisition of vital signs. Within an IEC, the CPA requires rapid, safe, and resource-efficient tools for initial sector allocation. We hypothesize that an initial assessment omitting vital signs significantly reduces triage time without compromising patient safety when combined with standardized chief complaints. To evaluate this, we utilized a modified ESI (mESI) linked with the presenting chief complaint.^5^, ^6^, ^7^

## 2 Methods

### 2.1 Study Design

The reporting of the design, development, and validation of the allocation model was conducted in accordance with the Transparent Reporting of a multivariable prediction model for individual Prognosis or Diagnosis + AI (TRIPOD+AI) guidelines, and the corresponding checklist is provided in the supplementary data upload.

A specific two-step triage workflow was prospectively implemented in the clinical routine before data extraction to ensure a strictly vital-sign-independent initial assessment. In the first step, triage nurses performed the modified ESI (mESI) assessment based solely on clinical evaluation and the CEDIS chief complaint without knowledge or prior measurement of vital signs. Immediately after this mESI documentation, a standard MTS assessment, including the measurement of all vital signs, was conducted as a secondary safety net for the individual patient. For this retrospective analysis, only the prospectively and blinded-assessed mESI scores from the first step were extracted, eliminating any risk of incorporation bias or confounding by vital parameters.

### 2.2 Setting

All ambulatory walk-in emergency patients from 2024 at the University Medical Center Schleswig – Holstein (UKSH) sites in Kiel and Lübeck were retrospectively extracted from the ED quality management system.

### 2.3 Selection of Participants

Patients from separate pediatric EDs were excluded (Ethics Vote, Christian-Albrechts-University Kiel D 633/25). After excluding patients who left the ED without medical contact, we randomly divided the remaining dataset into training (70%) and test (30%) sets.

### 2.4 Outcomes

Variables collected included modified ESI category (mESI) 1 to 5, CEDIS chief complaint, final case status (outpatient vs. inpatient), the need for an observation unit (OU) stay (defined as direct transfer to ICU, Intermediate Care, or stroke unit), and discharge reason. The mESI used here is the classic ESI variant without the provision for upgrading patients based on abnormal vital signs.^7^

### 2.5 Data Analysis

Mean outpatient rates (number of outpatient patients divided by the total number of patients in a group) were determined for every specific combination of chief complaint and mESI category in the training dataset. Subsequently, each patient was assigned the outpatient rate for their complaint-adapted initial assessment category.

The hypothetical allocation of patients to the ED or AOC followed these criteria: patients in mESI categories 1 and 2 were assigned exclusively to the ED as high-risk patients based on clinical impression, regardless of chief complaint. For patients in mESI categories 3 to 5, allocation was calculated using a theoretical outpatient rate based on the individual pretest probability for outpatient treatment.

The allocation was determined for outpatient rates from 0% to 100% in 1% increments, and the optimal threshold for outpatient allocation within mESI categories 3 to 5 was determined by maximizing the Youden Index (J), calculated as: J = Sensitivity + Specificity - 1. Mathematically, the Youden Index identifies the cut-off point on the Receiver Operating Characteristic (ROC) curve where the vertical distance to the diagonal reference line is maximized, giving equal weight to sensitivity and specificity. However, a major clinical limitation of using the Youden Index as a standalone metric in emergency care logistics is this exact mathematical parity. In emergency triage, a false-negative allocation (undertriage of an inpatient to the outpatient sector) carries a vastly higher risk of patient harm than a false-positive allocation (overtriage to the core emergency department).

To address this limitation and prioritize patient safety, the base algorithm was augmented with a predefined clinical safety override, resulting in the optimized L-mESI (L-mESI opt.).

The L-mESI opt. algorithm provides a binary allocation recommendation, assigning patients either to the core emergency department (ED) or to the colocated acute outpatient clinic (AOC). As illustrated in the sequential clinical flowchart (Fig 1), allocation follows a deterministic two-step approach. First, patients classified as mESI levels 1 or 2 are assigned directly to the ED. Second, for patients classified as mESI levels 3 to 5, the mESI category is combined with the CEDIS chief complaint. If a neurological high-risk symptom (acute headache or dizziness) is documented, the clinical safety override assigns the patient directly to the ED. For all remaining complaint-mESI combinations, allocation was based on the outpatient probability derived from the training dataset. Patients with an outpatient probability of ≥74%, corresponding to the maximum Youden Index, were allocated to the AOC, whereas patients below this threshold were allocated to the ED. The 74% threshold represents the baseline setting evaluated in the present study and may be adjusted for future implementation based on local diagnostic capacity and predefined acceptable levels of allocation risk.

**Figure 1:**
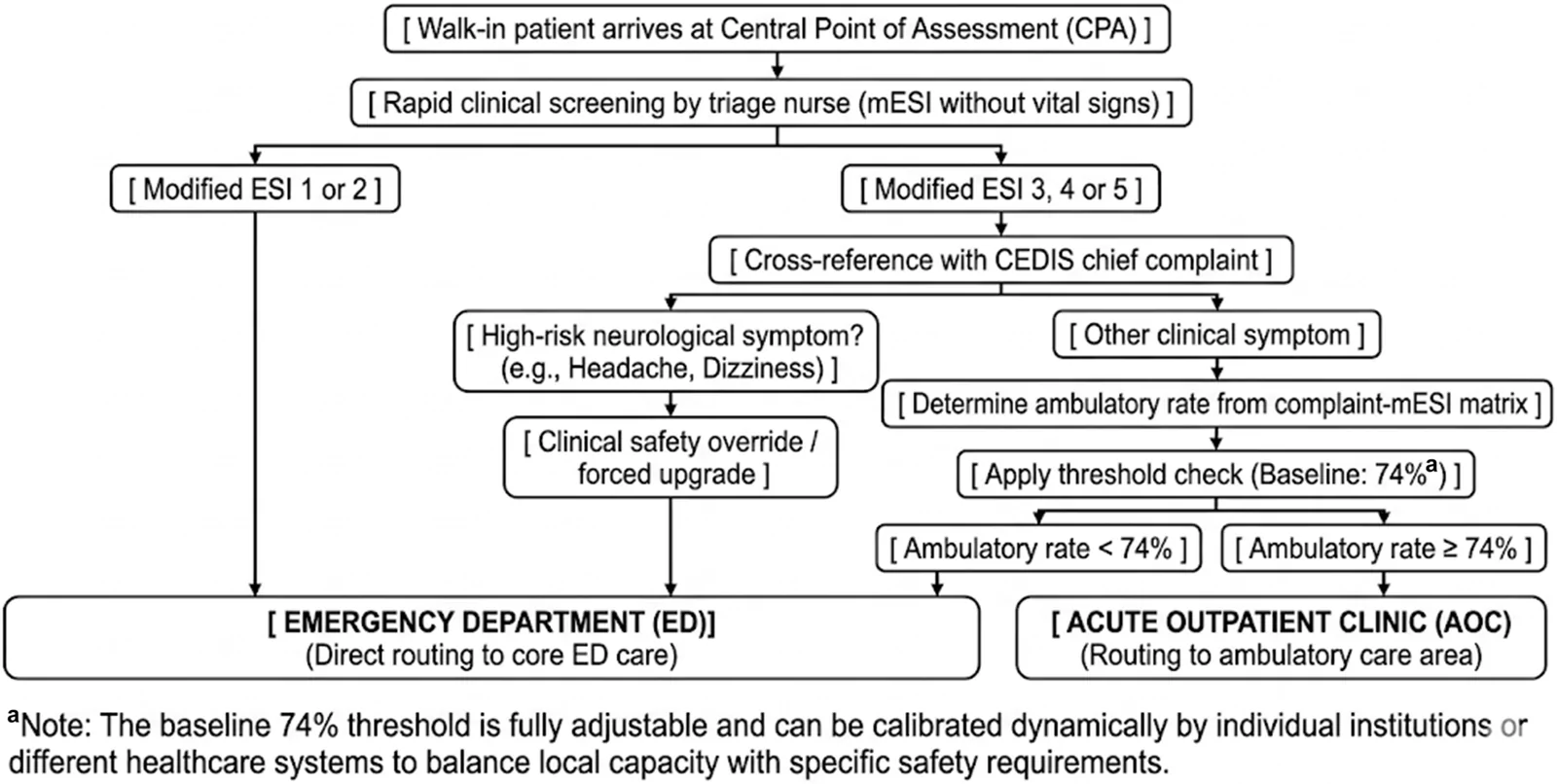
Patient allocation workflow as a binary recommendation to either the emergency department or the acute outpatient clinic.

The allocation results were compared against the actual patient case status (outpatient vs. inpatient) to create an error matrix. Additionally, the proportion of patients who required transfer to an OU or died in the real dataset despite AOC allocation was recorded. Finally, the allocation results were validated using the test dataset (Fig 2). The robustness of the estimates was tested using bootstrapping (Table S1).

**Figure 2:**
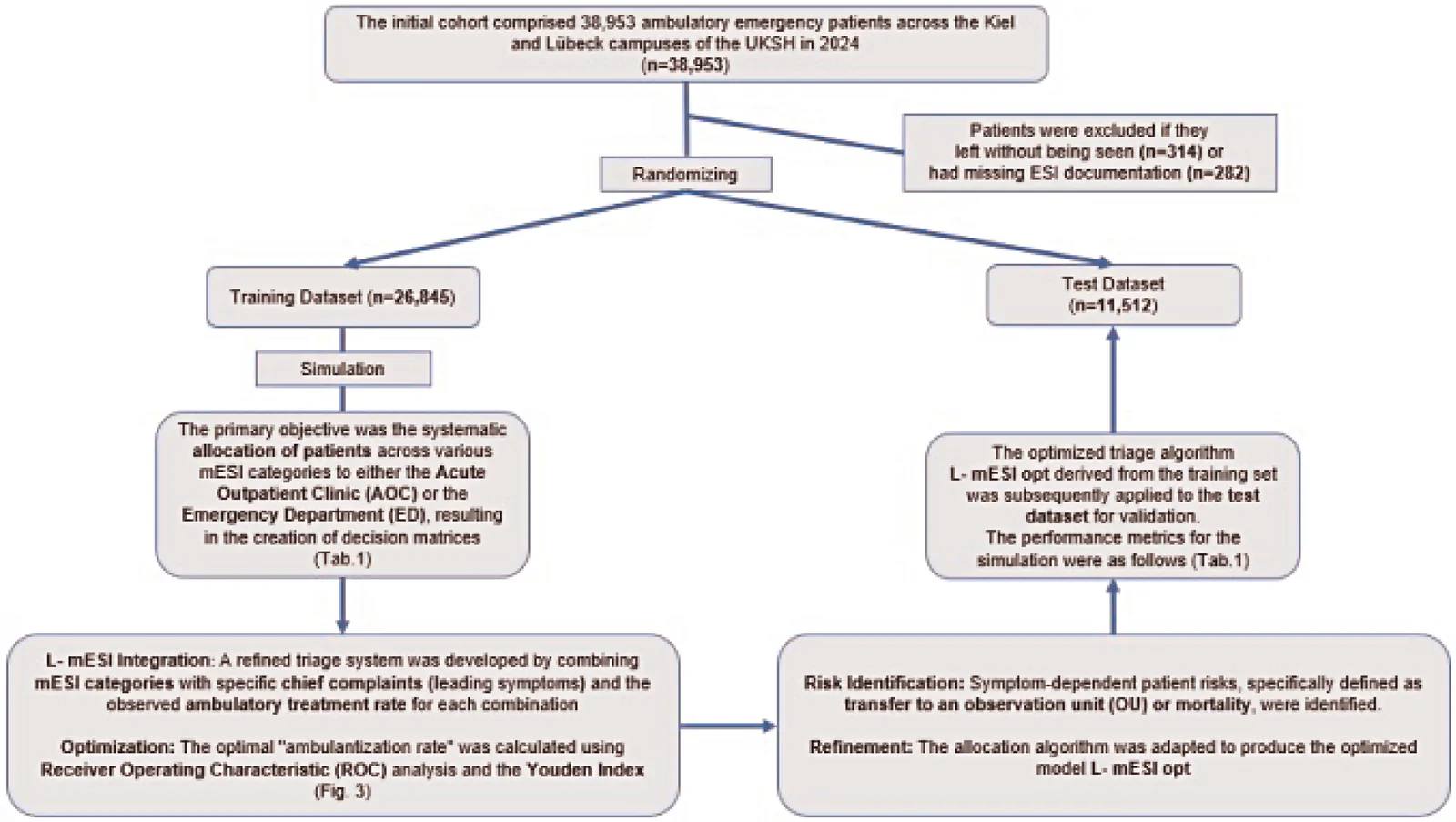
Presentation of study methodology and retrospective patient cohort of two German university EDs, Kiel and Lübeck, in absolute numbers of ambulatory emergency patients in 2024.

## 3 Results

### 3.1 Patient Cohort

In 2024, 38,953 walk-in emergency patients presented at the two UKSH sites. A total of 314 patients left the ED before being seen by a physician. For 282 patients, no mESI assessment was documented; therefore, we analyzed 38,357. Randomization resulted in 26,845 (70.0%) cases in the training dataset and 11,512 (30.0%) in the test dataset (Fig 1).

### 3.2 mESI Categories and Outpatient Rates

The patient distribution showed that mESI categories 3 and 4 were the largest groups. The rates of outpatient discharge (outpatient rates) for mESI categories 1 to 5 were 33.3%, 53.9%, 66.5%, 79.0%, and 89.8%, respectively (Fig 3).

**Figure 3:**
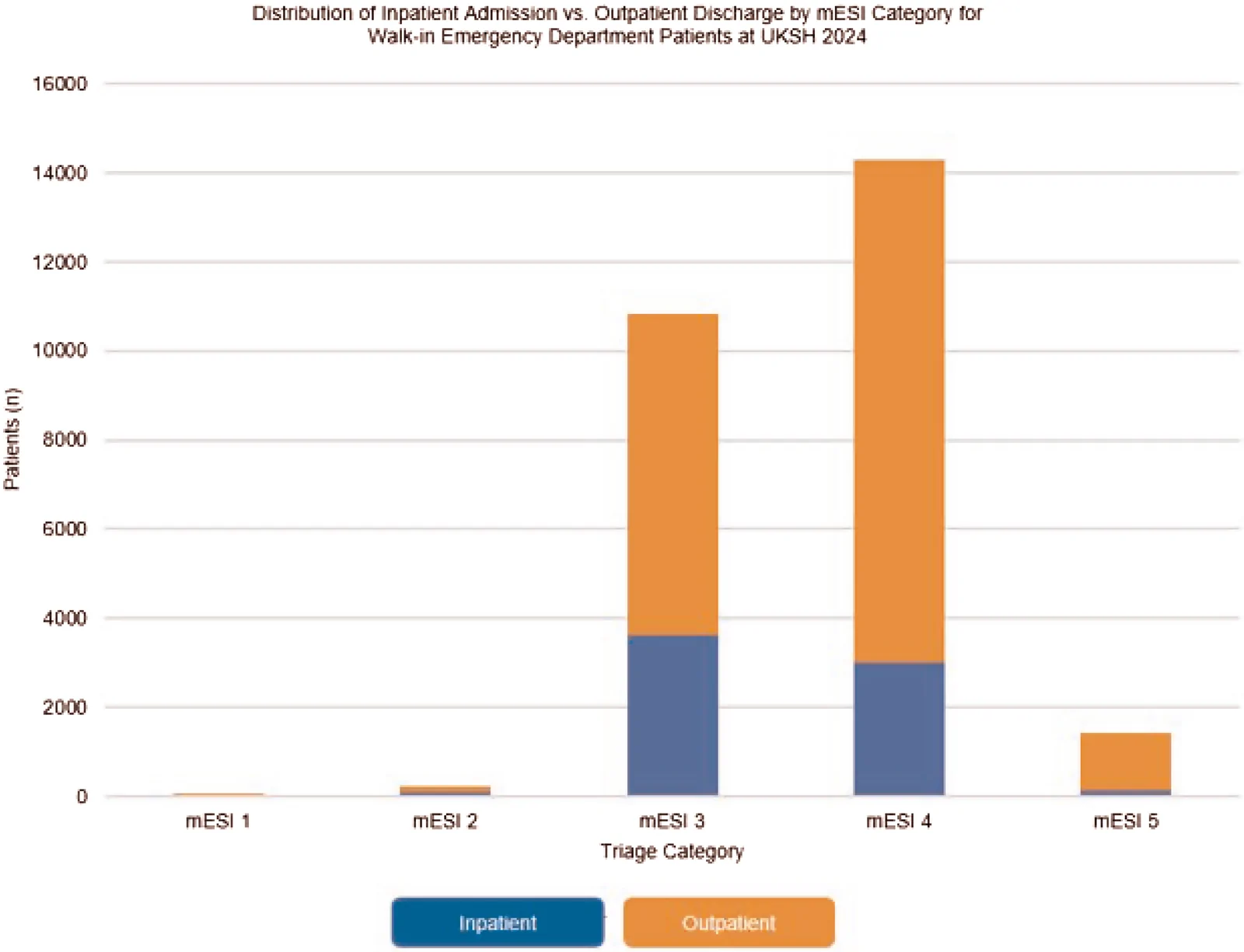
Distribution of case status (outpatient discharge vs inpatient admission) for walk-in emergency patients based on the determined modified emergency severity index (mESI) category.

The mean outpatient discharge rate of mESI 3 (66.5%) significantly limits its effectiveness as a standalone tool for safely distinguishing between outpatient and inpatient care needs. Table 1 displays the hypothetical distribution between the ED and AOC.

**Table 1:** Performance of the hypothetical allocation algorithms (L-mESI vs. standard mESI).

Allocation Categories	ESI 1-3 to ED/ESI 4-5 to AOC	ESI 1-2 to ED /ESI 3-5 to AOC	Modified L-mESI	Optimized L-mESI-opt (Training)	Optimized L-mESI-opt (Validation)
Total patients, n (%)	26,845 (100.0)	26,845 (100.0)	26,845 (100.0)	26,845 (100.0)	11,512 (100.0)
Allocated to ED, n (%)	11,136 (41.5)	291 (1.1)	10,898 (40.6)	12,233 (45.6)	5271 (45.8)
Allocated to AOC, n (%)	15,709 (58.5)	26,554 (98.9)	15,947 (59.4)	14,612 (54.4)	6241 (54.2)
TP: ED & Inpatient, n (%)	3780 (33.9)	144 (49.5)	4596 (42.2)	4898 (40.0)	2071 (39.3)
TN: AOC & Outpatient, n (%)	12,558 (79.9)	19,767 (74.4)	13,612 (85.4)	12,579 (86.1)	5,332 (85.4)
FP: ED & Outpatient, n (%)	7356 (66.1)	147 (50.5)	6302 (57.8)	7335 (60.0)	3200 (60.7)
FN: AOC & Inpatient, n (%)	3,151 (20.1)	6,787 (25.6)	2,335 (14.6)	2,033 (13.9)	909 (14.6)
Sensitivity %, (95% CI)	54.54 (53.36-55.71)	2.08 (1.77-2.44)	66.31 (65.19-67.41)	70.67 (69.58-71.73)	69.50 (67.82-71.12)
Specificity %, (95% CI)	63.06 (62.39-63.73)	99.26 (99.13-99.37)	68.35 (67.70-69.00)	63.17 (62.49-63.83)	62.49 (61.46-63.52)
PPV %, (95% CI)	33.94% (95% CI: 33.07%-34.83%)	49.48% (95% CI: 43.78%-55.20%)	42.17% (95% CI: 41.25%-43.10%)	40.04 (39.17-40.91)	39.29 (37.98-40.62)
NPV %, (95% CI)	79.94 (79.31-80.56)	74.44 (73.91-74.96)	85.36 (84.80-85.90)	86.09 (85.52-86.64)	85.44 (84.54-86.29)
**Safety Events in AOC**					
Transfer to OU, n (% AOC, 95% CI)	134 (0.85; 0.72-1.01)	389 (1.46; 1.33-1.62)	96 (0.60; 0.49-0.73)	37 (0.25; 0.18-0.35)	20 (0.32; 0.21-0.49)
Death in AOC, n (% AOC, 95% CI)	51 (0.32; 0.25-0.43)	128 (0.48; 0.41-0.57)	18 (0.11; 0.07-0.18)	17 (0.12; 0.07-0.19)	8 (0.13; 0.06-0.25)

AOC, acute outpatient clinic; ED, emergency department; ESI, emergency severity index; FN, false negative; FP, false positive; L-mESI, leading-symptom-weighted modified Emergency Severity Index; mESI, modified Emergency Severity Index; NPV, negative predictive value; OU, observation unit (ICU/IMC/stroke unit); PPV, positive predictive value; TP, true positive; TN, true negative.

### 3.3 Complaint-Specific Outpatient Rates

The calculation of outpatient rates adapted to complaint revealed high heterogeneity. The correlation between mESI category and outpatient rate varied by chief complaint (Table 2). For example, with "Shortness of breath," the rate increased with higher mESI categories as expected, whereas "Palpitations" showed an inverse trend. For "Abdominal pain," no clear correlation was observed.

**Table 2:** Different ambulatory rates (%) for select chief complaints according to the mESI category.

Chief Complaint	mESI 1	mESI 2	mESI 3	mESI 4	mESI 5
Abdominal pain (%)	N/A	63.6	58.0	68.4	79.3
Dyspnea (%)	N/A	30.0	38.3	48.4	100.0
Palpitations (%)	N/A	81.3	67.2	64.2	50.0

mESI, modified emergency severity index.

### 3.4 L-mESI Initial Assessment Procedure

ROC analysis using the L-mESI combinations in the training dataset yielded an area under the curve (AUC) of 0.71 and a maximum Youden Index (J) of 0.35 (Fig 3). Allocation to the AOC based on a 74% pretest probability (maximum Youden Index) resulted in 15,947 (59.4%) patients being referred to the AOC. However, 14.6% required subsequent ED admissions, leading to a final ED decompression of 50.7% of all walk-in patients.

The ROC curve illustrates the diagnostic performance of the L-mESI algorithm in determining the optimal threshold for outpatient care allocation among patients in mESI categories 3 to 5. Patients in mESI categories 1 and 2 were consistently allocated to the ED. The L-mESI model (blue markers) achieves an AUC of 0.71, demonstrating superior predictive value for inpatient treatment requirements compared with a nonoptimized approach. The diagonal grey line represents no discrimination (AUC = 0.50). We used the maximum Youden Index (J = 0.35) to identify the optimal cut-off point, balancing ED decompression with high patient safety. This threshold corresponds to a 74% outpatient pretest probability, optimizing the negative predictive value for critical clinical events.

### 3.5 Individual Complaint-Adapted Safety Refinement (L-mESI opt.)

Complaint-adapted triage using Youden Index optimization resulted in significantly fewer transfers to OUs than the standard mESI approach.

A detailed clinical review of the 96 AOC-assigned patients requiring OU transfer in the training set revealed a vulnerability of the base algorithm concerning neurological emergencies. Headache (n = 30) and dizziness (n = 29) accounted for most of these critical secondary transfers. The application of the clinical safety override for these specific symptoms in the L-mESI opt. reduced the rate of critical safety events in the AOC from 0.9% to 0.3%. The most compelling evidence for this method’s efficacy was the substantial increase in sensitivity for inpatient treatment, rising from 54.5% using the standard ESI to 69.5% (95% CI: 67.82 to 71.12) with the L-mESI-opt algorithm. Furthermore, this modification maintained significant ED decompression (46.9% workload reduction) while simultaneously reducing the rate of critical safety events in the AOC from 0.9% to 0.3% (n = 37), thereby underscoring the superior safety profile of this symptom-weighted patient steering logic (Fig 4).

**Figure 4:**
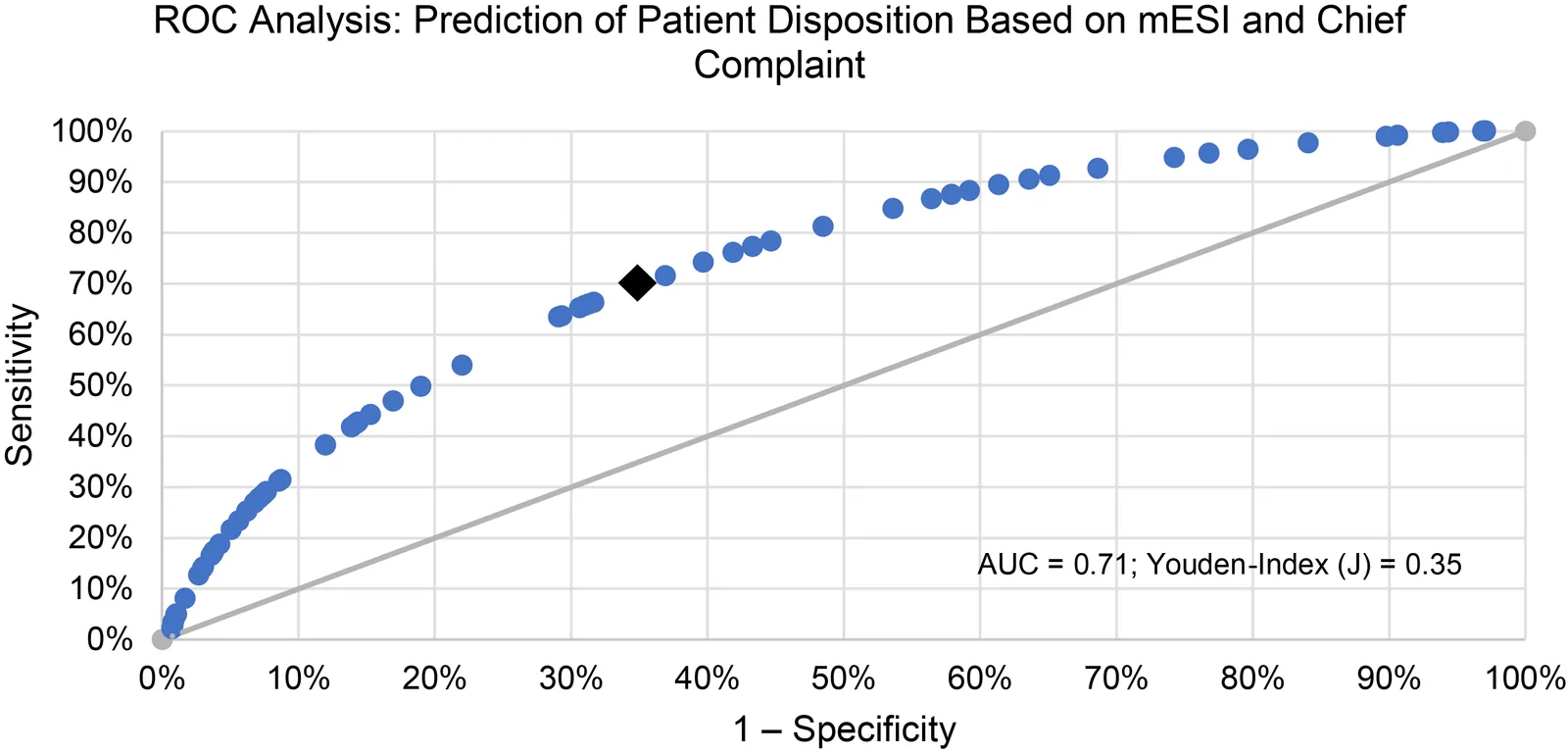
Receiver operating characteristic (ROC) analysis for patient allocation.

### 3.6 Validation of the Test Dataset

Validation confirmed that sensitivity and specificity remained consistent with minimal deviations, whereas ED decompression and patient safety remained high. Bootstrap analysis (5,000 resamples) confirmed higher sensitivity (71.14%, 95% CI: 68.62 to 73.72) and lower ICU/death rates for L-mESI-opt than mESI alone (Table S1).^8^

### 3.7 Symptom Distribution in the ED and AOC

After applying the L-mESI opt. the spectrum of chief complaints differed significantly between the two care sectors (*P* < .001). The 20 most frequent complaints in the ED were largely dominated by internal medicine and neurological symptoms (representing 65.2% of the ED top-20 cohort); the AOC mainly treated patients with musculoskeletal pain and minor trauma (representing 46.8% of the AOC top-20 cohort). This distinct distribution underscores the symptom-based allocation logic’s high discriminative power (Table 3).

**Table 3:** Top 20 CEDIS chief complaints by clinical allocation (ED vs AOC).

Rank	ED	n	%	AOC	n	%
1	Abdominal pain	2124	17	Lower-extremity pain	1481	10
2	Headache	891	7	Upper-extremity pain	1447	10
3	Shortness of breath (dyspnea)	743	6	Upper-extremity injury	1380	9
4	Direct referral/transfer	713	6	Back pain	910	6
5	Palpitations/Irregular heartbeat	633	5	Laceration /puncture/abrasion	704	5
6	Abnormal laboratory findings/imaging results	602	5	Lower extremity injury	680	5
7	Flank pain	585	5	Minor nonspecific complaints	613	4
8	Dizziness/vertigo	485	4	Not determined	534	4
9	Chest pain (cardiac)	461	4	Localized swelling/erythema	501	3
10	Sensory disturbance / paresthesia	401	3	Major trauma (blunt)	361	2
11	Extremity weakness /stroke symptoms	385	3	Urinary retention	335	2
12	Minor nonspecific complaints	343	3	Pregnancy problems (>20 weeks)	321	2
13	Not determined	340	3	Wound check/follow-up	289	2
14	General weakness	320	3	Scrotal pain or swelling	270	2
15	Fever	218	2	Vaginal bleeding	260	2
16	Localized swelling/erythema	216	2	Rash	242	2
17	Hematuria	194	2	Chest pain (noncardiac)	239	2
18	Visual disturbance	193	2	Earache	222	2
19	Syncope/presyncope	179	1	Bites (animal or human)	196	1
20	Rectal bleeding/melena	173	1	Hypertension	185	1

Percentages are rounded to the nearest whole number (training dataset).

AOC, acute outpatient clinic; CEDIS, Canadian Emergency Department Information System; ED, emergency department.

## 4 Limitations

A further notable limitation of our framework stems from the inherent subjectivity involved in assigning initial triage categories and chief complaints. Although both the ESI and the CEDIS taxonomy provide standardized criteria, their real-world application heavily relies on the individual clinical judgment, experience, and institutional culture of the intake triage staff. Variations in documentation habits and subtle differences in triage training across different hospital networks—or across international healthcare systems—can significantly influence how a patient’s initial presentation is captured. Because our data are derived from two major university medical centers, the specific baseline outpatient rates determined for individual complaint-mESI pairings may reflect regional operational practices. Consequently, external validation in diverse clinical environments is necessary to assess how robustly these symptom-weighted matrices perform when faced with different baseline triaging behaviors and multi-institutional cultures.

In addition, omitting vital signs accelerates risk assessment but may influence the upgrade of mESI categories 3 to 5. Future prospective studies must define the diagnostic capacity (laboratory, imaging) required in the AOC to prevent excessive back-transfers to the ED.

## 5 Discussion

### 5.1 Resource Prediction vs Clinical Urgency

To understand the clinical and administrative implications of our findings, it is vital to acknowledge the historical paradigm split between different emergency screening systems. Although the MTS was strictly developed as an acuity-based instrument to define immediate medical urgency, the ESI was never originally purported to be a pure triage system. Rather, for levels 3 through 5, the ESI functions as an institutional resource-prediction framework. Utilizing a resource-predictive score to guide binary routing decisions between a core ED and an outpatient clinic creates a profound operational tension. Maximizing administrative efficiency—accelerated in our model by omitting time-consuming mandatory vital sign assessments—directly challenges clinical risk management.

Because resource consumption does not linearly correlate with physiological deterioration, stable-appearing patients in the midacuity categories (mESI 3 to 5) inherently risk being misallocated to the outpatient sector, where unrecognized critical outcomes could occur. Although our validation data show that the actual clinical risk was low—with secondary intracenter transfers to observation units at just 0.3%—this structural tension underscores that mathematical optimization via the Youden Index alone is insufficient for clinical safety. A purely statistical cut-off treats sensitivity and specificity with equal mathematical parity, which is unacceptable when a false-negative allocation (undertriage to the outpatient clinic) carries vastly higher risks of patient harm than a false-positive allocation. This clinical reality is precisely why we augmented the base algorithm with an expert-rule clinical safeguard L-mESI opt). By overriding the statistical threshold for high-risk neurological chief complaints, we deliberately prioritized clinical safety over maximum administrative throughput, establishing a vital safety net against atypical, high-risk emergencies.^1^, ^2^, ^9^, ^10^, ^11^

Our error analysis identified a critical limitation of relying solely on an urgency score without vital signs for specific cohorts: the undertriage of neurological emergencies. Patients with critical neurological conditions, such as early stroke or intracranial hemorrhage, frequently present with isolated symptoms like headache or dizziness while maintaining stable vital signs. Our decision to categorically upgrade these symptoms in the L-mESI algorithm is strongly supported by existing literature. Royl et al^11^ demonstrated that headache and dizziness are among the most frequent neurological chief complaints in the ED, accounting for 20% and 12% of all neurological consults, respectively. Because these conditions are common and inherently difficult to differentiate from benign causes at the triage desk, incorporating them as explicit high-risk criteria into the symptom-based steering logic is a necessary clinical safeguard rather than a statistical artefact. This highlights the indispensable value of the CEDIS chief complaint in compensating for the omission of vital parameters. Our results demonstrate that mESI 3 patients—traditionally categorized as urgent —exhibit a 66.5% outpatient rate. This high degree of overtriage regarding resource consumption indicates that clinical urgency is not linearly correlated with the need for inpatient infrastructure.^12^, ^13^, ^14^ By integrating the chief complaint of CEDIS, the L-mESI opt. algorithm transcends the limitations of traditional ED triage by identifying symptom-specific patterns that allow for safe outpatient diversion, even when a patient is assigned a moderate urgency level.

This simulation study describes an initial assessment procedure for IEC based on a modified ESI combined with chief complaints. The primary advantages of this approach are the ease of parameter acquisition (mESI without vital signs) and the capacity for situational adjustment by varying the pretest probability for outpatient treatment.^7^, ^15^

The settings established via the Youden Index enable a workload reduction of approximately 50% for the ED while maintaining high patient safety. Although complete separation of outpatient and inpatient sectors is unfeasible—the ED retains its intersectoral character—the L-mESI opt. algorithm offers significant relief to overburdened EDs.^16^, ^17^, ^18^

When evaluating the 14.6% rate of AOC-allocated patients requiring subsequent ED admission (false negatives), this metric must be contextualized within the overall logistical impact. L-mESI opt. approximately 54% of all walk-in patients are diverted to the AOC. A 14.6% return rate from this diverted cohort means that ultimately, only about 8% of the total walk-in population experiences a secondary transfer back to the ED in another study from Hans et al^5^ at the University Freiburg with an IEC. Consequently, the ED still achieves a massive net reduction in patient volume of over 45%. In the context of severe ED crowding, this net decompression far outweighs the logistical friction caused by secondary intracenter transfers.

Slagman et al^3^ noted a 2.7% risk of undertriage with the Structured Initial Medical Assessment in Germany (“Strukturiete medizinische Ersteinschätzung für Deutschland” —SmED).^19^ Our potential patient risk was lower (0.3% OU transfer, 0.1% death). However, this system is only safe if the AOC is located within an IEC in immediate proximity to the ED.

Furthermore, our data confirm that patients referred to the AOC primarily present with trauma-associated symptoms. Therefore, AOCs and on-call practices should be equipped with the expertise and technology to treat trauma patients.^16^

### 5.2 Socio-Cultural Variations and International Generalizability

Beyond these inner-methodological trade-offs, the external generalizability of our findings must be interpreted within the specific context of the German healthcare system. Patient demographics, emergency care utilization patterns, and clinical documentation habits vary substantially across international borders. For instance, translating this algorithm to a US-American ED would introduce a fundamentally different patient population, characterized by varying baseline healthcare access, different insurance-driven presentation patterns, and a significantly higher proportion of non-Caucasian patients compared with our German cohort. These socio-cultural and systemic disparities could inherently shift baseline outpatient rates and impact algorithm performance.

However, the underlying operational logic of the L-mESI opt. framework is highly adaptable to international settings. Structurally, the colocated AOC described in this study directly mirrors established international models, such as an onsite “Fast-track” unit, a “Vertical care” system, or a colocated “Urgent care” center. A major operational bottleneck in international emergency medicine, particularly in the United States, is the crowding caused by ESI category 3 patients. Our simulation demonstrates that standard urgency scores alone poorly predict resource disposition, as mESI-3 patients exhibited a 66.5% outpatient rate. By integrating the standardized CEDIS chief complaint taxonomy, our algorithm can safely differentiate between true acute presentations and ambulatory-appropriate workflows within this critical cohort. Consequently, our model should not be viewed as a rigid blueprint, but rather as an intellectual catalyst for international providers to explore how symptom-weighted triage can optimize resource allocation and accelerate door-to-allocation times according to localized demographic, cultural, and institutional realities.

Crucially, the logistical acceptability of a 14.6% secondary transfer rate hinges entirely on the architectural and organizational structure of the acute care facility. This algorithm is explicitly designed for an IEC featuring a Joint Desk or CPA, where the ED and AOC are physically collocated. In such a setting, an upstream transfer back to the ED simply requires directing the patient down the hall, presenting a minimal logistical burden. Conversely, applying this algorithm to geographically separated care structures (eg, dispatching patients to distant on-call practices) would render a 15% return rate clinically and logistically unacceptable. Thus, spatial integration and shared IT infrastructure are the absolute prerequisites for the safe and efficient implementation of the L-mESI opt.

## Author Contributions

Domagoj Schunk and Sebastian Wolfrum: Conceptualisation, Methodology, Formal analysis, Investigation, Writing – Original Draft, Visualisation, Project administration, Writing – Review and Editing.

Milda Aleknonyte-Resch, Arvid Lepsien: Methodology, Resources, Data Curation, Data Analysis, Statistics, Validation, Review and Editing.

Alina Balandin, Jan Niclas Kersebaum, Hans Jörg Busch: Resources, data curation, review, and editing.

Florian Ketels, Sascha Thielke: Data analysis, statistics, review, and editing.

## Funding and Support

By *JACEP Open* policy, all authors are required to disclose any and all commercial, financial, and other relationships in any way related to the subject of this article as per ICMJE conflict of interest guidelines (see www.icmje.org). The authors have stated that no such relationships exist.

## Declaration of Generative AI and AI-Assisted Technologies in the Writing Process

During the preparation of this work the author Domagoj Schunk used ChatGPT (OpenAI) in order to check grammar, structure, and writing. After using this tool/service, the author reviewed and edited the content as needed and takes full responsibility for the content of the published article.

## Conflict of Interest

All authors have affirmed they have no conflicts of interest to declare.
