# Supplementary material for: Combining a Modified Emergency Severity Index Score with Chief Complaints - Validation of a New Allocation Algorithm for Integrated Emergency Centers
Source: J Am Coll Emerg Physicians Open. 2026 Nov 5;7(6):100534. doi: 10.1016/j.acepjo.2026.100534 (PMC13769126; doi:10.1016/j.acepjo.2026.100534)
Supplement: Supplementary file 1 [file mmc1.docx]

**Supplemental Material: Methodology and Data Analysis**

**"Combining a Modified Emergency Severity Index Score with Chief Complaints - Validation of a New Allocation Algorithm for Integrated Emergency Centres “**

**Study Design and Data Source**

This retrospective cohort study analyzed treatment data from all adult walk-in emergency patients (*n*=38,357) during the year 2024 at the Kiel and Lübeck campuses of the University Medical Center Schleswig-Holstein. Walk-in patients were identified via the "No transport" field within the clinical information system. Patients from separate pediatric emergency departments, as well as those who left the ED without medical contact ("Left without being seen") or did not receive an ESI assessment, were excluded. The cleaned dataset was randomly split into a training set (70% of cases) for model development and a test set (30% of cases) for internal validation.

To ensure a strictly vital-sign-independent initial assessment, a specific two-step triage workflow was prospectively implemented in the clinical routine prior to data extraction. In the first step, the triage nurses performed the modified ESI (mESI) assessment based solely on clinical evaluation and the CEDIS chief complaint, deliberately without knowledge or prior measurement of vital signs. Immediately following this mESI documentation, a standard Manchester Triage System (MTS) assessment, including the measurement of all vital signs, was conducted as a secondary safety net for the individual patient. For this retrospective analysis, only the prospectively and blinded-assessed mESI scores from the first step were extracted, eliminating any risk of incorporation bias or confounding by vital parameters."

**Definition of Predictors and Endpoints**

The modified Emergency Severity Index (mESI) category and the patient’s CEDIS chief complaint served as predictor variables for the algorithm. The modified ESI did not utilize vital parameters for further discrimination. The primary outcome parameter for model development was case status (outpatient vs. inpatient). To evaluate patient safety, secondary "safety" endpoints included transfer to an observation unit (OU)—defined as direct transfer to an intensive care unit, intermediate care unit, or stroke unit—as well as death within the emergency department.

**Algorithm Development (Training Set)**

The development of the algorithm (L-mESI) followed a three-step process within the training dataset:

1. **Calculation of Pre-test Probability:** First, we determined the empirical ambulatory rate (proportion of outpatient cases) for every combination of mESI category (1–5) and CEDIS chief complaint. This rate served as the complaint-adapted pre-test probability for ambulatory treatment.
2. **Development of the Base Algorithm (L-mESI):** We simulated a hypothetical allocation to the Emergency Department (ED) or the Acute Outpatient Clinic (AOC). As an initial safety rule, all patients in mESI categories 1 and 2 were categorically assigned to the ED. For all other patients (mESI 3–5), the individual pre-test probability (ambulatory rate) was used as a predictor. We identified the optimal allocation threshold by maximizing the Youden Index (Sensitivity + Specificity - 1) via Receiver Operating Characteristic (ROC) analysis.
3. Clinical Safety Refinement **(L-mESI-opt):** Rather than purely mathematical optimization, we conducted a structured clinical review of the false-negative cases from the base algorithm (i.e., AOC-allocated patients requiring ICU transfer). This qualitative analysis revealed a distinct clinical cluster for neurological symptoms, specifically Headache and Dizziness, were disproportionately represented among these critical cases. Because neurological emergencies often present with normal initial vital signs, an mESI-based assessment can systematically underestimate their urgency. To establish a robust safety net, these specific complaints were defined as neurological high-risk symptoms and integrated into the L-mESI-opt model as an absolute override, routing these patients directly to the ED [10].

**Validation and Robustness Testing**

The validation of the final L-mESI-opt algorithm was conducted in two stages:

- **Internal Validation (Test Set):** The ambulatory rates and the optimized allocation algorithm (including the threshold and high-risk symptoms) determined in the training set were applied to the independent 30% test dataset. Performance metrics (sensitivity, specificity, and patient safety) were calculated and compared with those from the training set.
- **Bootstrapping (Robustness Analysis):** To estimate the stability of the entire model development process, a non-parametric bootstrapping procedure (*B*=5,000 iterations) was performed on the full dataset. In each iteration, a bootstrap sample (stratified by site) was drawn as a new training set; the excluded cases ("out-of-bag") served as the test set. The complete three-stage model development process was re-executed for each of the 5,000 samples. Means and 95% confidence intervals (percentile method) for all metrics were calculated to confirm the robustness of the model selection [15].

### Table S1. Performance and Safety Metrics of Allocation Models (Bootstrap Analysis, B = 5,000)

|  | **ESI 1–3 to ED / ESI 4–5 to AOC** | **ESI 1–2 to ED / ESI 3–5 to AOC** | **L-mESI (Base)** | **L-mESI-opt (Safety Refinement)** |
| --- | --- | --- | --- | --- |
| Sensitivity, % (95% CI) | 54.02  (52.71–55.29) | 2.13  (1.75–2.50) | 67.72  (64.73–70.84) | 71.14  (68.62–73.72) |
| Specificity, % (95% CI) | 62.87  (62.13–63.58) | 99.28  (99.15–99.41) | 64.58  (61.29–67.19) | 60.47  (57.76–62.64) |
| PPV, %  (95% CI) | 33.63  (32.64–34.58) | 50.66  (44.37–56.81) | 40.00  (38.56–41.37) | 38.54  (37.37–39.67) |
| NPV, %  (95% CI) | 79.69  (79.01–80.38) | 74.44  (73.85–75.02) | 85.18  (84.35–86.01) | 85.75  (84.92–86.55) |
| Safety Events in AOC, % |  |  |  |  |
| Transfer to OU, % (95% CI) | 0.78  (0.63–0.94) | 1.43  (1.28–1.58) | 0.53  (0.32–0.75) | 0.29  (0.19–0.40) |
| Death in AOC, % (95% CI) | 0.33  (0.24–0.43) | 0.48  (0.39–0.58) | 0.12  (0.06–0.19) | 0.12  (0.06–0.19) |
| Absolute Case Counts  (Mean) |  |  |  |  |
| True Positives (TP) | 1,969.2  (1,900–2,035) | 77.5  (64–91) | 2,468.9  (2,350–2,599) | 2,593.5  (2,485–2,707) |
| True Negatives (TN) | 6,578.9  (6,473–6,687) | 10,389.4  (10,273–10,509) | 6,758.7  (6,408–7,045) | 6,327.9  (6,044–6,564) |
| False Positives (FP) | 3,386.0  (3,801–3,975) | 75.5  (62–89) | 3,706.2  (3,429–4,059) | 4,137.0  (3,904–4,429) |
| False Negatives (FN) | 1,676.3  (1,615–1,739) | 3,568.0  (3,483–3,656) | 1,176.6  (1,059–1,289) | 1,052.1  (957–1,148) |
| Absolute Safety Events |  |  |  |  |
| Transfer to OU (n) | 64.4  (52–77) | 199.8  (179–221) | 42.0  (25–61) | 21.4  (14–30) |
| Death in AOC (n) | 27.3  (20–35) | 67.4  (55–81) | 9.6  (5–15) | 9.0  (4–14) |

**Table Footnotes:** Data are presented as means with 95% confidence intervals (95% CI) derived from a non-parametric bootstrapping procedure (B = 5,000 iterations). Values are presented as means with 95% confidence intervals to demonstrate the stability of performance metrics, including sensitivity, specificity, positive predictive value (PPV), and negative predictive value (NPV). In each iteration, the complete three-stage model development process (threshold optimization via Youden Index and high-risk symptom identification) was re-executed on a stratified random sample to ensure the robustness of the performance metrics.

Abbreviations: ED, Emergency Department; AOC, Acute Outpatient Clinic; L-mESI, Symptom-weighted modified Emergency Severity Index; OU, Observation Unit (includes ICU, IMC, and Stroke Unit); PPV, Positive Predictive Value; NPV, Negative Predictive Value; TP, True Positive; TN, True Negative; FP, False Positive; FN, False Negative.
